# Reasons for disengagement from antiretroviral care in the era of “Treat All” in low‐ or middle‐income countries: a systematic review

**DOI:** 10.1002/jia2.26230

**Published:** 2024-03-17

**Authors:** Rachael M. Burke, Hannah M. Rickman, Clarice Pinto, Peter Ehrenkranz, Augustine Choko, Nathan Ford

**Affiliations:** ^1^ Clinical Research Department London School of Hygiene and Tropical Medicine London UK; ^2^ Malawi Liverpool Wellcome Clinical Research Programme Queen Elizabeth Central Hospital Blantyre Malawi; ^3^ Global HIV, Hepatitis and STIs Programme World Health Organisation Geneva Switzerland; ^4^ Global Health, Bill & Melinda Gates Foundation Seattle Washington USA; ^5^ International Public Health Department Liverpool School of Tropical Medicine Liverpool UK; ^6^ Centre for Infectious Disease Epidemiology and Research School of Public Health and Family Medicine Faculty of Health Sciences University of Cape Town Cape Town South Africa

**Keywords:** antiretroviral, care cascade, disengagement, loss to care, reasons, re‐engagement

## Abstract

**Introduction:**

Disengagement from antiretroviral therapy (ART) care is an important reason why people living with HIV do not achieve viral load suppression become unwell.

**Methods:**

We searched two databases and conference abstracts from January 2015 to December 2022 for studies which reported reasons for disengagement from ART care. We included quantitative (mainly surveys) and qualitative (in‐depth interviews or focus groups) studies conducted after “treat all” or “Option B+” policy adoption. We used an inductive approach to categorize reasons: we report how often reasons were reported in studies and developed a conceptual framework for reasons.

**Results:**

We identified 21 studies which reported reasons for disengaging from ART care in the “Treat All” era, mostly in African countries: six studies in the general population of persons living with HIV, nine in pregnant or postpartum women and six in selected populations (one each in people who use drugs, isolated indigenous communities, men, women, adolescents and men who have sex with men). Reasons reported were: side effects or other antiretroviral tablet issues (15 studies); lack of perceived benefit of ART (13 studies); psychological, mental health or drug use (13 studies); concerns about stigma or confidentiality (14 studies); lack of social or family support (12 studies); socio‐economic reasons (16 studies); health facility‐related reasons (11 studies); and acute proximal events such as unexpected mobility (12 studies). The most common reasons for disengagement were unexpected events, socio‐economic reasons, ART side effects or lack of perceived benefit of ART. Conceptually, studies described underlying vulnerability factors (individual, interpersonal, structural and healthcare) but that often unexpected proximal events (e.g. unanticipated mobility) acted as the trigger for disengagement to occur.

**Discussion:**

People disengage from ART care for individual, interpersonal, structural and healthcare reasons, and these reasons overlap and interact with each other. While HIV programmes cannot predict and address all events that may lead to disengagement, an approach that recognizes that such shocks will happen could help.

**Conclusions:**

Health services should focus on ways to encourage clients to engage with care by making ART services welcoming, person‐centred and more flexible alongside offering adherence interventions, such as counselling and peer support.

## 1 INTRODUCTION

Since 2015, WHO has recommended that people living with HIV be offered antiretroviral therapy (ART) irrespective of clinical staging or CD4 count [1]. The rollout of this “Treat All” recommendation and scale‐up of ART services has individual and community benefits: reduced mortality and increased life expectancy of individuals living with HIV and reduced HIV transmission [2, 3, 4]. Many countries have achieved or are making progress towards achieving the 95‐95‐95 goals of 95% of people living with HIV knowing their status, 95% of those diagnosed with HIV having started ART and 95% of those who started ART to be virologically suppressed [2].

Initiating ART and attaining virological suppression represent only the first milestones in a continuous, life‐long process. For ART to be effective, people living with HIV need to consistently engage with health services, and health services need to be consistently accessible to people who use them. Disengagement from HIV care—having started taking ART, but then stopped treatment for a defined period of time—is relatively common: in a large multi‐country study which updated data through tracing, only 67% of people who started ART between 2009 and 2014 were engaged in care at 5 years [5], with similar findings in more recent studies in Zambia, South Africa, Tanzania and eSwatini [6, 7, 8, 9]. People initiating ART at clinics are often re‐initiating treatment after a period of disengagement, although clients do not always report previous ART experience: a recent review found that 20−50% of ART patients who present for ART were re‐initiators [10]. Disengagement from ART care jeopardizes individuals and communities from realizing the benefits of “Treat All” [3, 11].

We conducted a systematic review to describe reasons why people disengage from HIV care in the era of treat all in low‐ and middle‐income countries. The objective was to identify interventions that may improve engagement in care and to provide recommendations for the design and conduct of future studies.

## 2 METHODS

We systematically reviewed the literature for studies which reported reasons for people disengaging from ART care during the era of treat all.

### 2.1 Definitions

Our working definition of disengagement from HIV care was having started taking ART, but then not taken ART for 30 days or more, consistent with published definitions [11]. Disengagement is distinct from lost to follow up (LTFU): LTFU is from the perspective of the health system and may include people who have disengaged from care, but may also include misclassifications such as unreported death, silent transfers (people who may appear lost from one clinic but are engaged in care elsewhere) and duplicate charts for a person within a single clinic. Because not all studies used the same definition of disengagement, we took a pragmatic approach to whether the participants included in studies were likely to be considered disengaged from care applying our definition. A “reason” for disengagement was defined as something which was stated in the paper as being a cause of disengagement as reported by a participant.

### 2.2 Search strategy

We used a highly sensitive search strategy with terms for HIV, ART and terms related to disengagement (including words such as disengage, retain, interruption, re‐initiate and others) and searched Medline and EMBASE using OVID (see Supplementary Appendix). We additionally hand‐searched abstracts from International AIDS Society Conferences from 2015 to 2022; abstracts from the Conference on Retroviruses and Opportunistic Infections are included in EMBASE.

### 2.3 Inclusion and exclusion criteria

We searched for studies published between 1st January 2015 (the start of “Treat All”) and 7th December 2022 that reported reasons for disengaging from care. We included studies where researchers had been in contact with and conducted interviews or surveys with adults, adolescents or children who had experienced disengagement from ART care. Participants could be disengaged at the point of study recruitment, or re‐engaged in care following a prior period of disengagement.

We did a two‐stage title and abstract screening where irrelevant papers were removed in the first step (this step excluded papers that were from high‐income settings, assessed planned treatment interruptions or did not include individuals on ART); papers meeting exclusion criterion at closer title/abstract review were removed in the second step (this step excluded papers that were done pre “Treat All” or did not report having followed up with disengaged individuals). We then did a full‐text review to identify studies meeting inclusion and exclusion criteria. Title and abstract screening was done by one author (RMB), with a subset of 10% of decisions checked by a second author (HR). Two authors independently screened full‐text articles to decide on inclusion. Discrepancies were resolved by consensus, including a discussion with a third author (NF).

Studies which included both people with and without experience of disengagement from care were included if reasons for disengagement were disaggregated by disengagement status or if more than half the participants had experienced disengagement. Studies were excluded if they reported barriers to care among people who nonetheless managed to stay engaged or compared baseline characteristics between people who did and did not engage, but did not report reasons for disengagement.

The study population was limited to people disengaging from care after “Treat All” had been implemented in the study country (approximately 2016 onwards in most countries), or for studies focusing on pregnant women, after “Option B+” policies were adopted (approximately 2010 onwards). We used this limitation because prior to “Treat All,” people starting ART, systems delivering it and community context were very different, limiting applicability to the current context. When CD4‐based eligibility criteria were in place, care delivery and counselling models served as gatekeepers, preventing some people from receiving services until they became eligible, and leading to pre‐ART loss to care; delaying care also resulted in people being sicker at the point of ART initiation. Antiretroviral drugs in common use in this period were associated with a greater frequency of side effects.

We included studies in low‐ or middle‐income countries according to World Bank definitions. We included both qualitative studies (in‐depth interviews or focus group discussions) and quantitative studies (using a survey or questionnaire), and studies in any age group or HIV risk group. There were no language restrictions. We excluded studies which primarily focused on adherence to ART in people who remained in clinical care. Detailed inclusion and exclusion criteria are in Supplementary Material. Studies limited to sub‐population with a clear reason for transitioning between care (e.g. moving from prison to community services) were excluded.

### 2.4 Data extraction and synthesis strategy

After reviewing the included papers, we categorized types of reasons using an inductive approach and discussion among authors to achieve consensus and ensure consistency. For all papers, we extracted data on country, population, number of participants, method of recruitment, definition of disengagement, method of determining reasons (in‐depth interviews or surveys), all reasons stated and (where available) what proportion of people reported each reason and whether any framework for categorizing reasons was used by each paper. We grouped results by population group: general populations of all adults disengaged from ART care, women who started ART when pregnant and key or other populations.

We assessed the quality of evidence using a modified Newcastle Ottawa Scale and a modified Critical Appraisal Skills Programme (CASP) toolkit (Supplementary Appendix). An overall “score” was not calculated because this could be misleading given the variety of study designs.

## 3 RESULTS

**Figure 1:**
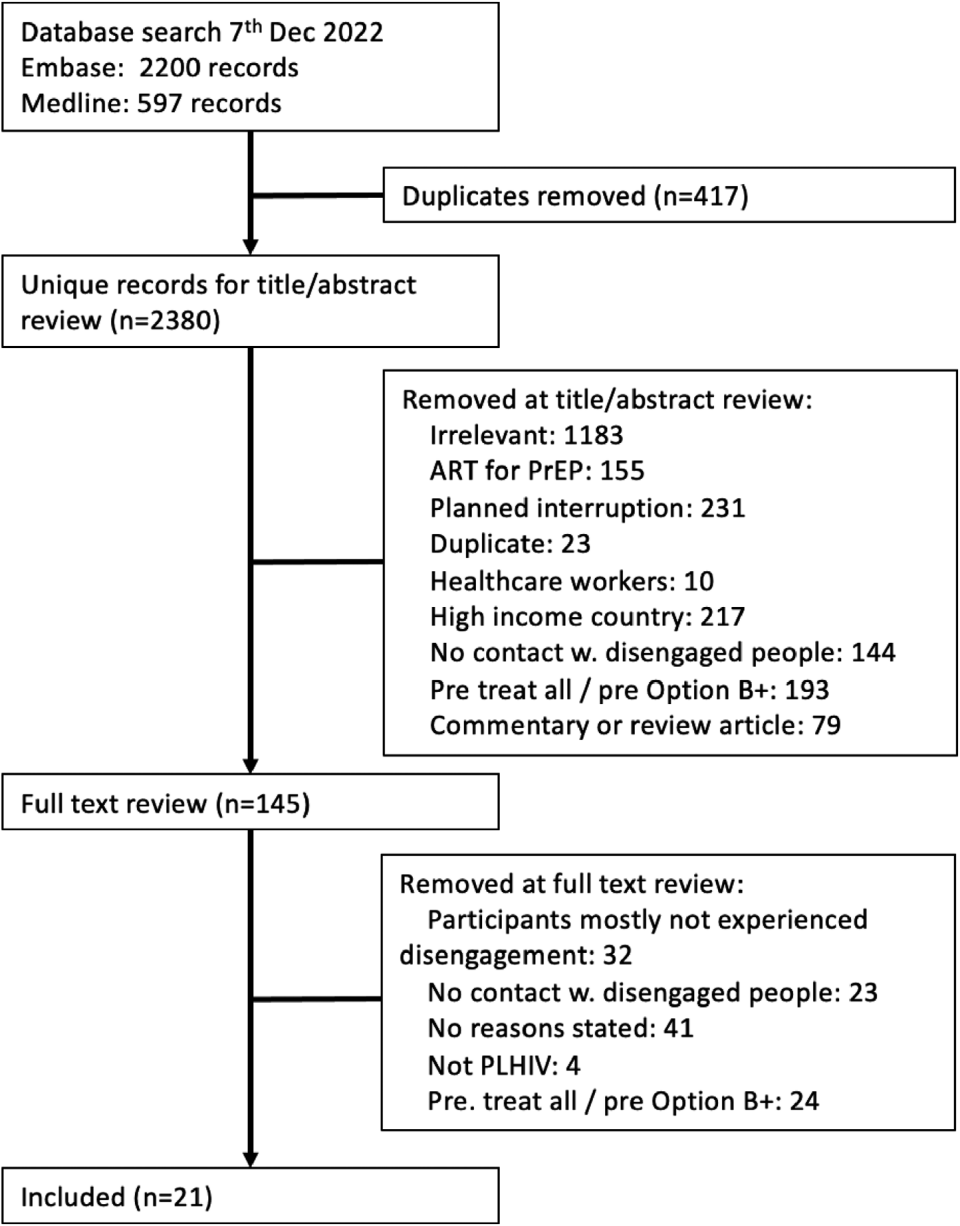
Study selection.

The database search yielded 2380 unique records, of which 145 records were reviewed as full text and 21 studies met all eligibility criteria and were included in the final review (Figure 1). Among the 21 studies, three were conference abstracts [12, 13, 14] and the remainder were full papers (Table 1). Six studies were about general adult populations [12, 14, 15, 16, 17, 18], nine were about women who started ART while pregnant [19, 20, 21, 22, 23, 24, 25, 26, 27] and six were about various smaller populations (people who use drugs [13], indigenous people in an isolated area [28], adolescents [29], gay/bisexual/other men who have sex with men [30], men only [31] and women only [32]). No studies included children. While all studies included people with experience of disengagement from ART, four studies included only participants who had subsequently re‐engaged with care [15, 17, 21, 32], and seven included a mixture of people who had re‐engaged after a period of disengagement and those who remained disengaged [14, 24, 26, 27, 28, 30, 31]. Twenty were single‐country studies with 16 in Africa, three in the Americas and one in Europe. One study was conducted in 13 African countries. The number of included disengaged participants per study ranged from 6 to 5008 people. Nine studies had quantitative surveys [12, 13, 15, 16, 19, 20, 21, 32, 33] (including studies where this was done alongside in‐depth interviews) and 14 studies included at least some information about how frequently various reasons were reported by participants [12, 13, 14, 15, 16, 17, 19, 20, 21, 22, 23, 24, 31, 32].

**Table 1:** Description of included studies and reasons for disengagement

Study	Country/year study conducted	Study design	Participants (number) Recruitment	Definition of disengagement
**General group adult PLHIV**				
Bisnauth et al. 2021 [15]	South Africa (2019−2020)	Questionnaire and in‐depth interview	General population (quantitative survey *n* = 562; in‐depth interview *n* = 30) Identified at point of re‐engagement	Self‐reported to be reinitiating ART, or already appeared on TIER.net national electronic client monitoring system (in context of a “welcome back” campaign)
Kafeero. 2020 [12]	Uganda (2019)	Questionnaire	General population (*n* = 5008) Traced from clinic records	“Categorized as lost to follow up” (no further information)
Chamberlin et al. 2021 [17]	Malawi (2017)	In‐depth interview	General population (*n* = 44) Clinic records reviewed to identify people with previous missed appointment	Missed an ART appointment (> 14 days late) but came back to care within 60 days
Nsoh et al. 2021 [16]	Cameroon (2019)	Interview with closed questions and in‐depth interview	General population (survey *n* = 271; in‐depth interview *n* = 13) Not described	> 30 days of stopping ART
Shabalala et al. 2018 [18]	Eswatini (2014–2016)	In‐depth interview	General population (*n* = 10) Traced from clinic records	Stopped ART refill for 3 months of longer from date of last appointment
Popoola et al. 2023 [33]	Thirteen African countries (2019−2010).^a^	Telephone or face to face questionnaire	General population (*n* = 430) Recruitment not described	“PLHIV who have ever disengaged from care” (no further information)
**Pregnant or postpartum women (including Option B+)**				
Atanga et al. 2017 [22]	Cameroon (2013−2014)	Not stated	Pregnant women (*n* = 36) Traced in community	>1 week late for an appointment and declined to come back when traced
Gugsa et al. 2017 [23]	Malawi (2015)	In‐depth interview	Pregnant women (*n* = 25) Traced from clinic records	Missed scheduled appointment by more than 21 days without telling hospital staff
Hoffman et al. 2017 [19]	Malawi (2014−2015)	Questionnaire	Pregnant women (*n* = 50) Traced from clinic records	Out of ART for >60 days (Malawi national definition of default)
Kiragga et al. 2021 [20]	Uganda (2017−2018)	Tablet‐based survey	Pregnant women (*n* = 37) Traced from clinic records	Non‐attendance of 6‐week post‐partum visit by 10 weeks after estimated date of delivery. Were contacted by study and declined to return
Kisigo et al. 2020 [24]	Tanzania (2017−2018)	In‐depth interview	Pregnant women (*n* = 12) Traced from clinic records, includes some back in care	Report of skipping medication in for at least 30 days in a 3‐month period, missing three consecutive monthly visits or high viral load at 6 months post‐partum
Kiwanuka et al. 2018 [25]	Uganda (2017)	In‐depth interview		Non‐attendance for 60 days from last visit
Nalubega et al. 2021 [26]	Uganda (2020)	In‐depth interview (via phone)		Had not attended 6 week post‐partum visit by 10 weeks after estimated date delivery
Sariah et al. 2019 [27]	Tanzania (2017)	In‐depth interview		Non‐attendance at clinic for > = 180 days
Sasse et al. 2022 [21]	Malawi (2015−2019)	Questionnaire with free text		Previously started first‐line ART, (usually Option B+ in a previous pregnancy) but not on ART at the time of HIV test in current pregnancy and no ART for at least 3 weeks
**Key population or other study with restricted inclusion criteria**				
Aguilera‐Mijares et al. 2022 [30]	Mexico, (not stated when)	In‐depth interview	Gay/bisexual/other men who have sex with men (*n* = 22) Traced from clinic records, includes some back in care	>3 months from last missed visit (disengaged)
Cecchini et al. 2019 [32]	Argentina (2016−2018)	Check box questionnaire plus free text	Women of childbearing age (*n* = 90) Traced in from clinic records, returned to clinic, recruited at point of re‐engagement	Three or more missing pharmacy pickups in the past 6 months or not attended physician visit past 12 months and reports not currently taking ART. All participants agreed to come back to care
Coursey et al. 2022 [31]	Malawi (2016−2017)	In‐depth interview	Men (*n* = 21) Traced from clinic records, includes some back in care	> = 14 days late for an appointment in the first 12 months on ART (including if had spontaneously returned)
Enane et al. 2021 [29]	Kenya (2019−2020)	In‐depth interview	Adolescents (*n* = 42) Identified in clinic records, traced and interviewed in community	> = 60 days late for last scheduled visit
Kandlen 2020 [13]	Russia (2016–2019)	Not reported	General population (high‐prevalence key populations) (*n* = 4375) Recruitment not stated (all clients from an NGO which supports PLHIV not in care)	Missed ART for 3 months, or for pregnant women or neonates, any missed appointment
Gabster et al. 2022 [28]	Panama (2019)	In‐depth interview	Indigenous people in isolated area (*n* = 13) Traced from clinic records, includes some people back in care	Missed at least two clinic visits within past 2 years

*Note*: Studies are by alphabetical order of first author, within each category.

Abbreviation: ART, antiretroviral therapy.

^a^ Countries were Uganda, South Africa, Nigeria, Ethiopia, Rwanda, Zambia, Eswatini, Lesotho, Kenya, Malawi, Mozambique, Zimbabwe and Sierra Leone.

Study quality varied considerably. Issues included unclear reporting of methods, particularly around recruitment, questionnaires or survey instruments used with no indication of validity or very low numbers of participants (i.e. fewer than 15 people). See the Supplementary Appendix for further details.

### 3.1 Conceptual framework

**Figure 2:**
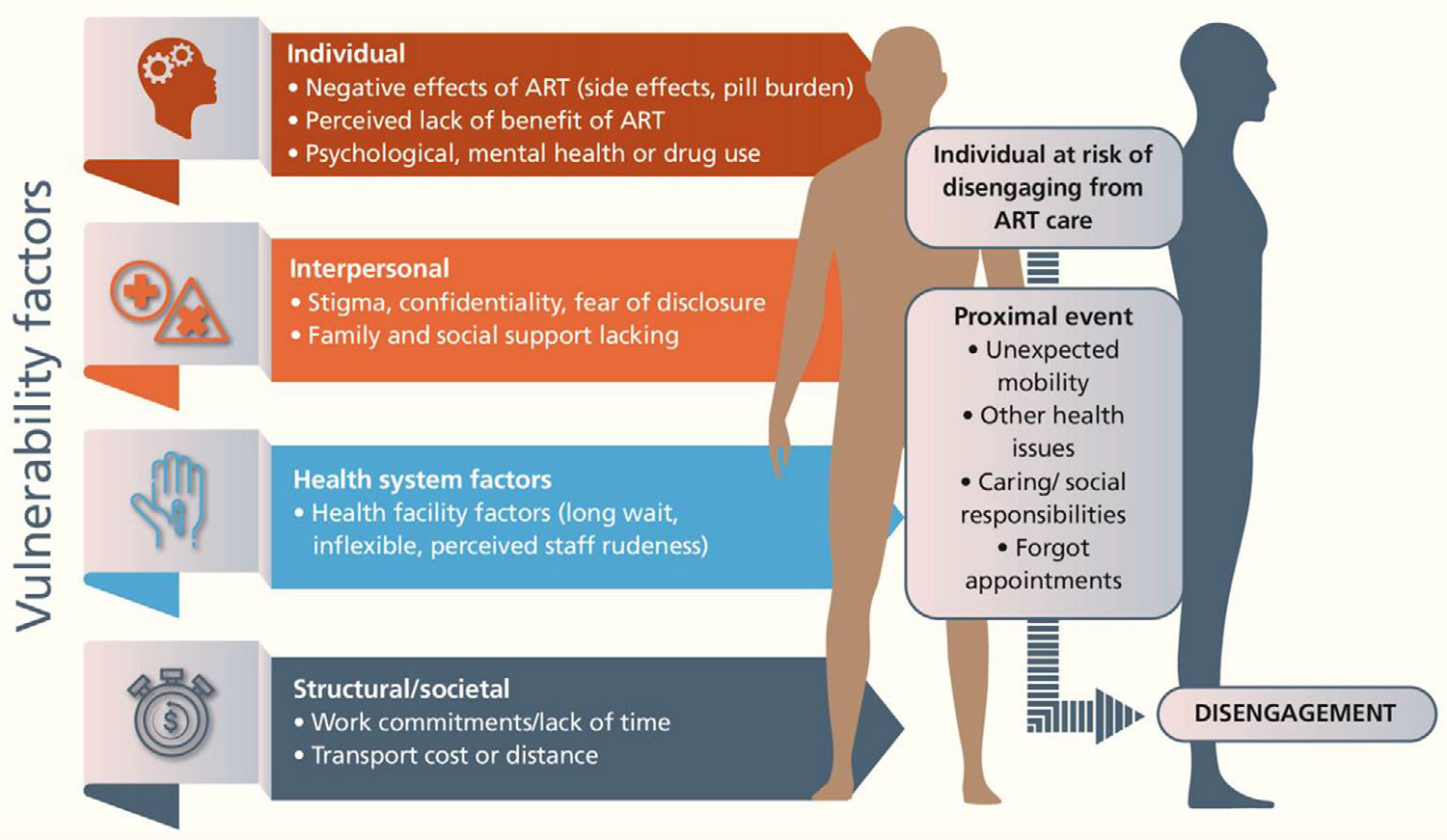
Conceptual framework for reasons for disengagement.

Thirteen studies reported a conceptual framework for understanding engagement in care, whether in a diagram or text. We used a social‐ecological model combining aspects of these frameworks, a framework from an earlier (before Treat All) systematic review [34], and induction from reasons in studies to identify individual, interpersonal, structural and healthcare facility‐related reasons for people to be vulnerable to disengagement from ART care (Figure 2). Individual‐level reasons included ART side effects or tablet issues, lack of perceived benefit of ART and psychological factors. Interpersonal factors related to stigma and perceptions of lack of confidentiality and lack of family or social support. Structural reasons were socio‐economic and health facility related. Many studies reported reasons for disengagement related to a proximal unexpected event such as unexpected mobility, often on the background of vulnerability to disengagement and we considered these reasons in a separate category.

**Figure 3:**
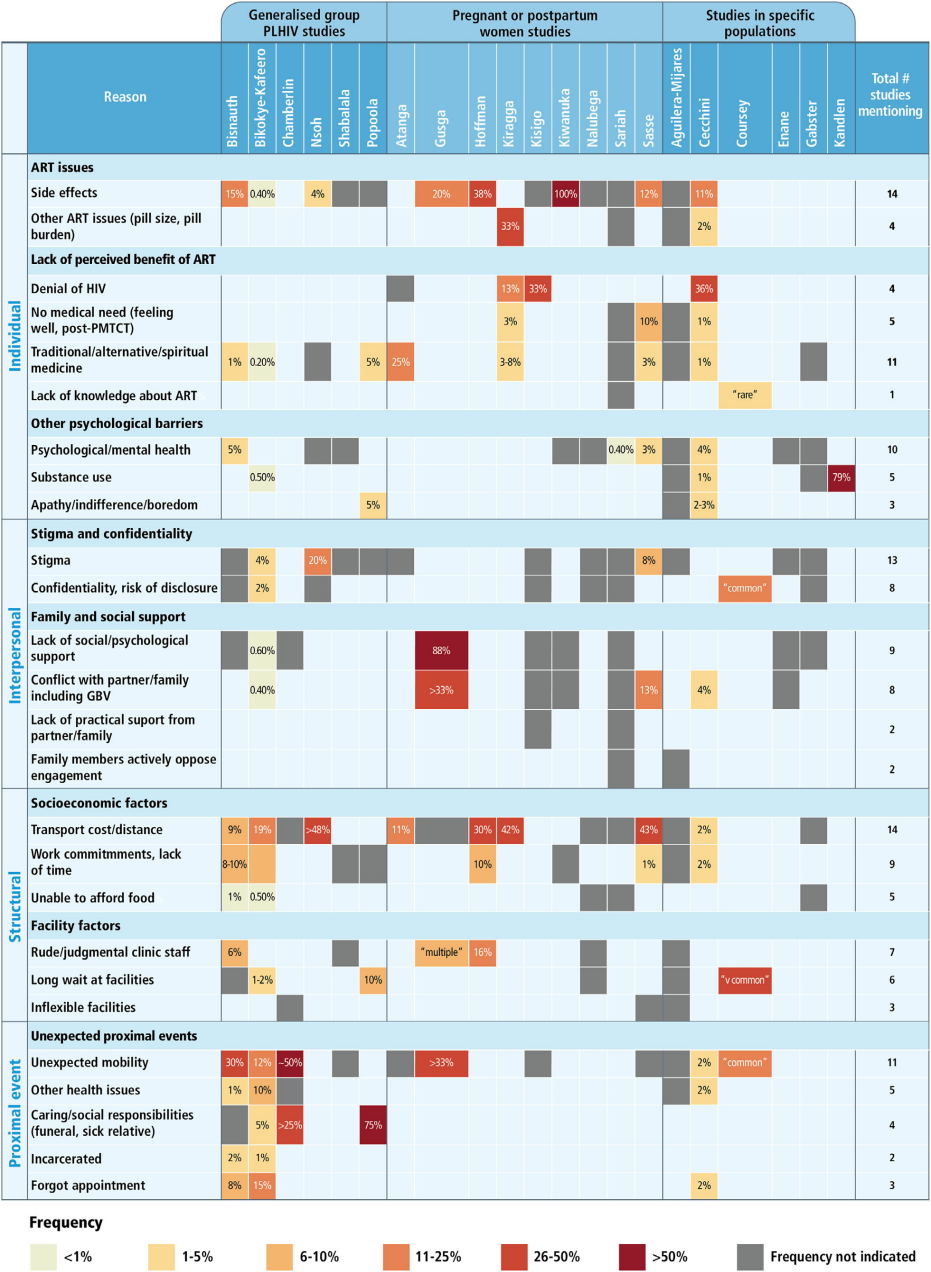
Aggregated reasons for disengagement reported by each study. GBV, gender‐based violence. Figure shows reasons, inductively grouped (right‐hand column). Shaded boxes indicate the reason was reported by the study. Where reported, the frequency of respondents mentioning the reason is indicated as a %. Where no % or other text is reported, the paper did not report a frequency of this reason.

Figure 3 summarizes reasons reported across the 21 studies and, where this was reported, indicates the proportion of participants reporting each reason.

#### 3.1.1 Individual reasons

Reasons related to ART medication were reported in 15 studies and included side effects (14 studies) and pill burden or pill size (four studies). Lack of perceived benefit to ART was reported in 13 studies, including denial of HIV status (four studies), desire to use traditional or religious medicine instead of ART (11 studies) and feeling well (five studies). Psychological distress or mental health reasons were reported in 11 studies, drug use in five studies and boredom/apathy/fatigue in three studies. Perceived stigma or concerns about perceived lack of confidentiality were reported in 14 studies.

#### 3.1.2 Interpersonal reasons

Interpersonal reasons for disengagement included ack of family and social support (interpersonal reasons) including lack of psychosocial support (nine studies) and conflict with partner or family including fear of gender‐based violence (eight studies).

#### 3.1.3 Structural/health facility reasons

Structural socio‐economic factors included transport costs or distance (14 studies) and work commitments or lack of time (nine studies). Structural health facility factors included rude or judgemental facility staff (seven studies), long waiting times (six studies) or inflexible schedules at facilities (four studies).

#### 3.1.4 Proximal events

Proximal events acting as acute precipitants of disengagement were reported relatively frequently. Eleven studies reported that unexpected mobility or need to travel was the immediate reason from disengaging from care. Health issues meaning a person was unable to attend clinic were reported in five studies and unexpected social commitments or other personal challenges were reported in four studies.

Among the four largest studies (>100 respondents), the main reported reasons for disengagement included individual (forgetfulness, perceived lack of need, side effects, active substance use), interpersonal (social responsibilities, conflict with partner) structural (transport, mobility) and health systems related (long waiting times) reasons [12, 13, 21, 33]. Among studies reporting quantitative data, the most frequently reported reasons within each study were acute precipitants (often mobility), lack of perceived benefit to ART (often denial of HIV status) and structural socio‐economic factors (often transport).

## 4 DISCUSSION

People disengage from HIV care for a variety of reasons and usually for multiple reasons which can occur together or over time. We identified reasons operating at individual, interpersonal, structural/social and healthcare system levels which increased an individual's risk of future disengagement from care. We found that in addition to these “vulnerability factors,” many studies reported a proximal trigger for disengagement: often an acute precipitant, such as unplanned travel for work or family reasons, and this occurred on a background of other factors that contributed to an underlying vulnerability to disengagement. We specifically restricted our review to disengagement during the “treat all” era; however, many of our findings—particularly acute events acting as a precipitant for disengagement have been reported in earlier (pre‐treat all) studies [34, 35].

Drivers of disengagement from care are complex and reasons overlap and interact. For example, the perceived lack of confidentiality at healthcare centres might interact with interpersonal concerns about HIV stigma, while a lack of family support to provide money for transport might interact with the structural issue of distance to the clinic and the cost of a bus journey. Reasons can overlap with and reinforce each other such that a chain of events that lead to disengagement where any one reason may be necessary but not sufficient (e.g. a cascade of experiencing trauma leading to isolation [29], depression and avoidance [32], or an unexpected family emergency leading to needing to travel combined with an unwelcoming health system and fear of being scolded on return [17]).

We were largely unable to meaningfully summarize results by population group (general adult population, pregnant women and specific populations). We observed that acute precipitants were mentioned less often in studies of pregnant women (three out of nine studies in pregnant women), compared to the general population or specific populations (mentioned in five out of six studies in general adult populations and three out of six in specific populations). However, in the one study that reported the frequency of reasons among pregnant women, acute precipitants were reported by one‐third of women—that is they were common reasons for disengagement among women in that study.

Sometimes reasons may contribute to difficulties engaging in care, but be neither necessary nor sufficient on their own. For example, mobility was often reported by people as an acute precipitant reason for disengagement; but a study in Mozambique showed people who reported travelling for work in the past 12 months were no more likely than people who had not travelled to have had gaps in picking up or taking ART (i.e. disengagement followed by re‐engagement) [36]. Mental health problems including depression were commonly reported reasons for disengagement, but a study from South Africa that recruited adolescents both engaged and disengaged from care and measured psychosocial stressors found that there was no difference comparing those engaged and disengaged in terms of prevalence of depressive symptoms or low psychosocial support [37]. Unfriendly healthcare staff or fear of being shouted at by healthcare staff was cited as a reason for disengagement by several studies; nevertheless, one study reported that while participants had experienced unfriendliness from clinic staff, this was not a reason for them to disengage [17]. By contrast, a cluster‐randomized intervention in Zambia to improve client experience at health centres did show an increase in engagement (but not viral suppression) [38].

Because so many reasons overlap, addressing one reason in isolation may not be enough to meaningfully reduce disengagement at the programme level. Nevertheless, while no single intervention is likely to result in sustained engagement of individuals at risk of disengagement, many of the cited reasons why individuals disengage should be addressed as part of service quality. Prescribing ART with minimal side effects as once‐daily fixed‐dose combinations to reduce pill burden, improving treatment literacy, providing social and psychological support, reducing HIV‐related stigma and ensuring that healthcare services are accessible, and provided in a non‐judgemental way are all recommended by WHO as part of routine service provision.

Traditionally, the HIV care continuum has been represented as a linear cascade. In order to better capture disengagement and re‐engagement, a cyclical cascade has been proposed that highlights that people can and do exit and return to care at various stages of the cascade [11]. Many of the included studies recruited people where all or some participants had re‐engaged in care, either spontaneously or after tracing. While the included studies are not representative prospective studies, it highlights the issue that periods of disengagement followed by re‐engagement in care are common in people living with HIV.

### 4.1 Challenges to reviewing disengagement

We encountered several challenges in this review. Different investigators use different words to describe similar concepts, including (dis)engagement—the preferred term for this review—LTFU and retention (or lack of retention), and these concepts overlap with missed appointments and suboptimal adherence. Different definitions are used by different studies, often to mimic national lost to follow‐up definitions and protocols for tracing, but sometimes as chosen by the researcher. While our definition (>30 days without ART) is clear and client‐centred, it can be hard to determine from clinic records which are based on attendance at clinic visits and pharmacy pick‐ups. Other studies and reviews have adopted other definitions of engagement [39]. Studies often combined results from groups of people with experience of disengagement and those who had remained in care (we included these papers if more than half people had experience of disengagement), and in some cases, definitions of disengagement were overly permissive (e.g. 14 days late to appointment). This means that some participants in studies in this review may not have met our criteria for disengagement.

When considering reasons, different researchers understandably used different words for similar concepts (such as “isolation” vs. “low social support”) and it was not possible to combine even the quantitative surveys in meta‐analysis as they had asked about different potential reasons. Of the studies which used quantitative surveys or questionnaires, the questionnaire development and validation were generally not well described and different studies asked about different potential reasons on questionnaires meaning data could not be aggregated. More standardized, validated questionnaires may help to more systematically capture reasons for disengagement in the context of future research or programmatic monitoring.

ART side effects were relatively commonly mentioned—and have been identified as important in earlier reviews of disengagement [40]—but no studies reported what ART regimen was being used. Between 2015 and 2019, WHO recommended first‐line ART was tenofovir‐lamivudine‐efavirenz, although some older studies (particularly pregnant women “Option B+”) may have used other regimens. From 2019, most programmes used tenofovir‐lamivudine‐dolutegravir (TLD) which is generally well tolerated. From 2019 onwards, programmes have also increasingly used models of ART delivery that include extended refill durations which help mitigate transportation and convenience challenges. Since these changes (TLD and multi‐month dispensing) only appeared in 2019, combining barriers reported before and after 2019 may not be valid.

A further limitation is that none of the studies were on younger children, and only one included adolescents [29]. While we grouped results according to studies in the general population, pregnant women and in specific populations, we were unable to draw specific conclusions about group‐specific reasons for disengagement. In general, the same types of reasons were reported across most studies (regardless of population group) and we were unable to do any meta‐analysis on frequencies of reasons because it was not possible to aggregate data from different papers.

All the participants in the studies had either re‐engaged with care following an episode of disengagement, or had been successfully contacted by a research team and had consented to participate in the study. The voices of people who disengage from care, do not re‐engage and either cannot be contacted or are unwilling to participate in research are, therefore, not represented. This may include more vulnerable individuals who are in greatest need of support, and as such it is critically important to understand their challenges to engaging in care.

## 5 CONCLUSIONS

Complex phenomena like the interaction between clients and health services over many decades can be difficult to summarize and generalize. Reasons for disengagement are necessarily individual to each person, and even reasons experienced by a group of people are likely to be specific to geographic or social communities rather than applying in all places and among all groups of people across time. Countries are moving closer to achieving 95‐95‐95 targets, but this is not a static goal, and disengagement contributes to ongoing transmission, illness and death associated with advanced HIV disease. It is, therefore, essential for HIV programmes and policy‐makers to understand the context‐specific factors which may promote or jeopardize engagement. This may be facilitated by a systematic collection of reasons for disengagement among those who disengage or re‐engage with HIV services.Developing a valid questionnaire tool that could be used for research and implementation would be useful.

Nevertheless, despite the broad range of contexts included in this review, some reasons were common across a number of studies and may act as a starting point for future efforts to reduce disengagement. Programmes could improve counselling and education about taking ART, including on U = U messaging, educating about side effects and emphasizing that while side effects may still be experienced on TLD, they are often temporary. Counselling about adherence should involve talking with clients to identify possible barriers and ensure that these are addressed using a person‐centred approach, which could mean the need for additional ART education, or addressing mental health, socio‐economic or other individual issues. Another frequently reported contributing factor was transport costs or distances. Ameliorating this problem is highly context‐dependent but offering community‐based decentralized ART options [41] or social protection to offset the cost of transport might be appropriate in some settings. Psychological distress and low social support were commonly mentioned as reasons for disengaging in this review; and trial and implementation science projects investigating the effectiveness of providing service navigation and social support to men in Malawi [42] and in South Africa [43] are ongoing. Additionally, many studies reported that acute events and unplanned travel precipitated disengagement; programmes should focus on increasing the flexibility of appointments if one is missed, procedures for getting an emergency supply of ART from an alternative facility to the client's usual clinic and communicating to clients about what to do if an appointment is missed or travel is necessary. Simplifying transfer processes and “welcome back” efforts are also critical. For all clients, clinics should be friendly, respectful and welcoming spaces. Using peer supporters/expert clients [44], and virtual tools to communicate with clients (such as SMS or WhatsApp messaging) [45] may be helpful in promoting engagement.

Further research on causes of disengagement could use or further develop our social‐ecological model (Figure 1), summarizing individual, interpersonal, structural and healthcare vulnerability factors and the role of acute proximal to design and implement appropriate interventions targeted to address the most acute retention needs in a given context. For example, in a concentrated epidemic, a programme might focus engagement efforts on sex workers or men who have sex with men. In a generalized epidemic, a programme might choose to put additional resources for psychosocial counselling or community ART delivery models in the highest prevalence geographic area. Further targeting could be done to address the needs of demographic groups known to have low retention rates in a particular setting. In an era of flatlined resources for HIV but ambitious goals to decrease morbidity, mortality and new infections, HIV programmes and providers must work to ameliorate the barriers faced by people living with HIV, with the first step being addressing issues related to the way care is delivered. Where the resilience of people with HIV can be overcome by an unexpected event, it is the responsibility of the health system to provide systems of care delivery that are supportive and flexible enough to absorb the repercussions.

## COMPETING INTERESTS

All authors declare no competing interests.

## AUTHORS’ CONTRIBUTIONS

NF conceived the review. RMB undertook the literature search and data extraction, with the support of HMR. All authors provided critical input in the interpretation of the data and approved the final version of the manuscript.

## FUNDING

This study received funding the World Health Organisation Global HIV, Hepatitis and STI programmes. RMB and HM are supported by Wellcome and PE by the Bill and Melinda Gates Foundation.

## Supporting information


**Appendix**: Reasons for disengagement from antiretroviral care in the era of “treat all” in low‐ or middle‐ income countries: a systematic review

## Data Availability

All data are contained in the manuscript and appendices.
