# Supplementary material for: Reasons for disengagement from antiretroviral care in the era of “Treat All” in low‐ or middle‐income countries: a systematic review
Source: J Int AIDS Soc. 2024 Mar 17;27(3):e26230. doi: 10.1002/jia2.26230 (PMC10945039; doi:10.1002/jia2.26230)
Supplement: Supplementary file 1 — Appendix: Reasons for disengagement from antiretroviral care in the era of “treat all” in low‐ or middle‐ income countries: a systematic review [file JIA2-27-e26230-s001.docx]

**Appendix to “Reasons for disengagement from antiretroviral care in the era of “treat all” in low- or middle- income countries: a systematic review”.**

**Contents:**

1. Search strategy
2. Detailed inclusion and exclusion criteria
3. Conceptual frameworks used in included papers
4. Study Quality Assessment
5. PRISMA checklist
6. References appendix

**1. Detailed search strategy**

Search terms used to search Medline. Search ran (using Ovid) on 7^th^ December 2022.

| Concepts related to HIV | 1. exp HIV Infections/  2. exp HIV/  3. exp HIV Long-Term Survivors/  4.HIV.tw.  5. hiv1.tw.  6. hiv2.tw.  7. "human immunodeficiency virus".tw.  8. "human immunedeficiency virus".tw.  9. "human immuno-deficiency virus".tw.  10. "acquired immunodeficiency syndrome".tw.  11. "acquired immunedeficiency syndrome".tw.  12. "acquired immune-deficiency syndrome".tw.  13. "acquired immuno-deficiency syndrome".tw.  14. AIDS-virus*.tw.  15. (human-immun* and "deficiency virus").tw.  16. (acquired immun* and "deficiency syndrome").tw.  17. 1 or 2 or 3 or 4 or 5 or 6 or 7 or 8 or 9 or 10 or 11 or 12 or 13 or 14 or 15 or 16 |
| --- | --- |
| Concepts related to treatment | 18. treatment*.tw.  19. therap*.tw.  20. exp Therapeutics/  21. 18or19or20  22. 17 and 21 |
| Concepts related to anti-retrovirals | 23. exp Anti-Retroviral Agents/  24. *Anti-Retroviral Agents/pd [Pharmacology]  25. exp Antiretroviral Therapy, Highly Active  26. Antiretrovir*.tw.  27. anti-retrovir*.tw  28. ARV.ti.  29. ART.ti.  30. HAART.tw.  31. anti-HIV.tw.  32. anti-human-immunodeficiency.mp.  33. anti-acquired-immuno-deficiency.mp.  34. anti-human-immuno-deficiency.mp.  35. anti-human-immune-deficiency.mp.  36. anti-human-immunedeficiency.mp.  37. anti-acquired-immunodeficiency.mp.  38. anti-acquired-immunedeficiency.mp.  39. anti-acquired-immune-deficiency.mp.  40. 23 or 24 or 25 or 26 or 27 or 28 or 29 or 30 or 31 or 32 or 33 or 34 or 35 or 36 or 37 or 38 or 39 |
| HIV & treatment, or ART | 41. 22 or 40 |
| Concepts related to (dis)engagement | 42. cessation.ti.  43. interrupt.ti.  44. return*.ti.  45. stopping.ti  46. withdraw*.ti.  47. disengag*.ti.  48. reengag*.ti.  49. re-engag*.ti.  50. re-initiat*.ti.  51. reinitiate*.ti.  52. restart*.ti.  53. re-start*.ti.  54. discontinu*.ti.  55. retain*.ti.  56. retention.ti.  57. interruption.ti.  58. 42 or 43 or 44 or 45 or 46 or 47 or 48 or 49 or 50 or 51 or 52 or 53 or 54 or 55 or 56 or 57 |
| Combining HIV/treatment OR ART & disengagement terms. | 58 and 41. |

**2. Detailed inclusion and exclusion criteria**

A full protocol can be obtained by emailing corresponding authors, the review protocol was not pre-registered.

*Inclusions*

- Adults and children who have experienced disengagement from ART care. Includes people who remain disengaged (e.g. identified through tracing), those at the point of re-engaging in care (e.g. people attending ART clinics to re-start ART) or people who have already re-engaged.
- Must be identified / re-engaging after “treat all” policies were adopted or, for studies of people starting ART while pregnant of breast feeding, after Option B+ policies were adopted in the country of the study.
- Must be in a low or middle income country using World Bank criteria.
- Must contain information to extract related to people’s stated reasons for disengagement (“reasons” can include “barriers” or other words that cover broadly the same content).
- Published in a peer reviewed journal, a conference proceedings / abstract indexed by EMBASE, or at the International AIDS Society Conference.

*Exclusions*

- Studies that only have baseline information on people who then disengage or are lost to follow up (i.e. do not contact dis-engagers).
- Not primary research (e.g. systematic reviews)
- Study does not contain reasons for disengagement available.
- Studies which only measured the prevalence or magnitude of an attribute (such as depression scores or distance from healthcare facilities) in people disengaged from care were excluded unless there was an indication that the study participants considered the attribute as the reason for their disengagement.
- Studies reporting treatment disengagement/interruption for medical reasons (e.g. planned treatment interruptions or serious side effects of treatment).
- Studies reporting reasons for non-adherence without disengagement from care.
- Studies in sub-population with a clear reason for transitioning between care (e.g. moving from prison to community services), **except** studies related to pregnancy, delivery and breastfeeding (i.e. studies related to pregnancy, delivery and breastfeeding will be included).

**3. Conceptual frameworks used in included studies**

If an included study had a conceptual framework we extracted information about how they framed the issue.

| **Paper** | Diagram / model / conceptual framework |
| --- | --- |
| Bisnauth et. al. PLoS One 2021 *(1)* | Findings have been organised into **health service** and **patient factors**. Health service findings are reported based on the Accessibility framework and its three components; availability; affordability and acceptability. **Changing life circumstances** was the most prominent reason for disengagement from care. |
| Chamberlin et. al. 2021 *(2)* | 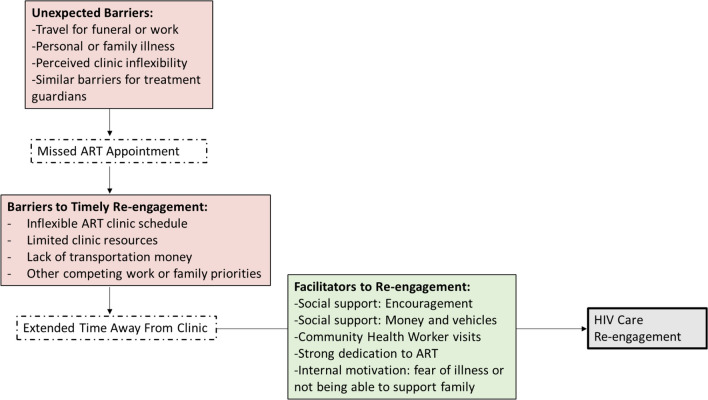 |
| Nsoh et. al. 2021 *(3)* | Client  Health system and care provider  Community-related |
| Shabalala et. al. 2018 *(4)* | The narrative analysis revealed how a **chain of events** had triggered step‐by‐step their decisions to discontinue treatment.  Initially most respondents reported mobility – relocating residence – as the main reason for discontinuing ART. Further probing revealed more complex circumstances: sub‐optimal care from health care providers, severity and prolonged medication side effects, fear of stigma, lack of food and social networks were entwined in the **process that leads to loss to follow‐up.** In‐depth analysis of individual cases revealed several intersecting reasons that occurred consecutively, as a **“chain of events”** |
| Kisigo et. al. 2020 *(5)* | 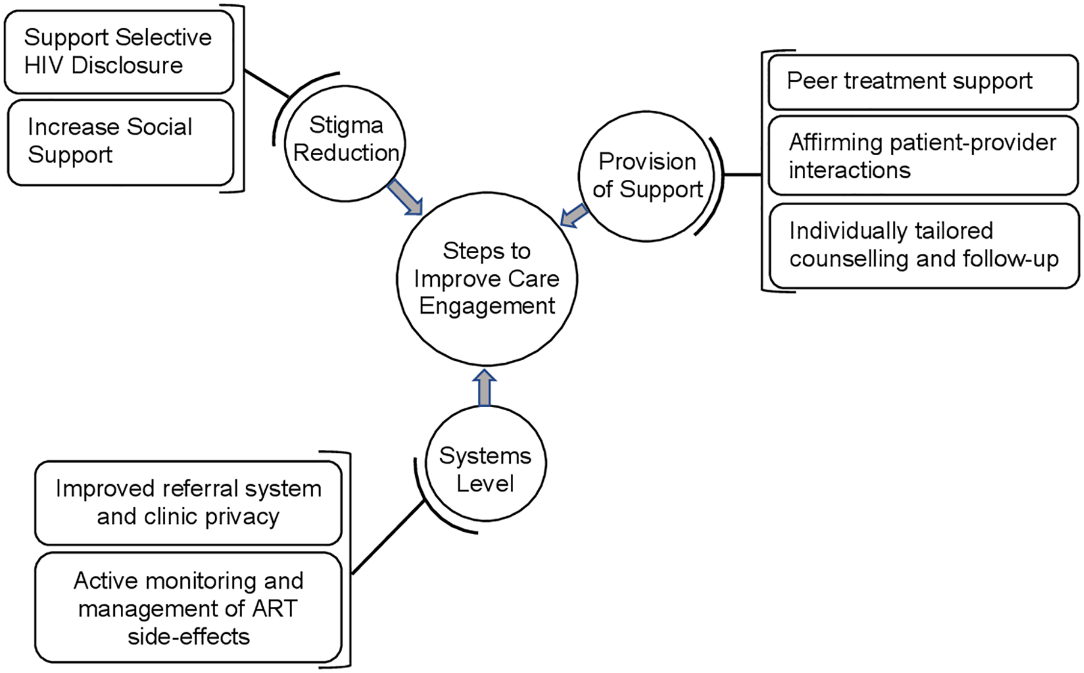 |
| Nalubega et. al. 2021 *(6)* | “Various barriers to retention in HIV care and to adherence to HIV medications have been reported, and generally categorised under three types namely; **structural** (e.g. transport difficulties, accessibility of healthcare facilities and limited finances), **clinical** (e.g. clinic delays, negative attitudes/experiences with healthcare personnel, clinic delays and fear of drug side effects) and **psychosocial** (e.g. HIV stigma, disclosure and poor family support) barriers [[7](https://bmcinfectdis.biomedcentral.com/articles/10.1186/s12879-021-06684-6#ref-CR7)]. These barriers tend to be similar across the African region as presented in published literature.” |
| Sariah et. al. 2019 *(7)* | **Table 4:** Emerged categories and main theme from the study  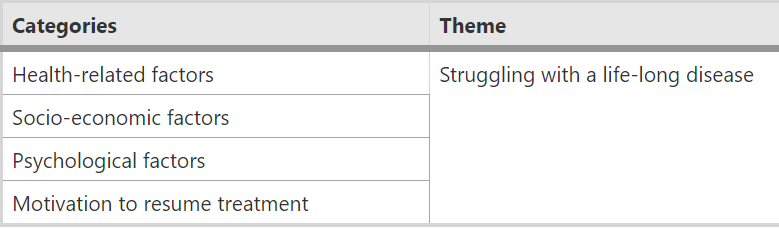 |
| Sasse et. al. 2022 *(8)* | 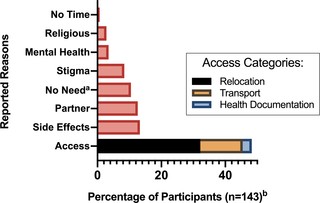 |
| Aguilera-Mijares et. al. 2022 *(9)* | ECOLOGICAL MODEL – intrapersonal, interpersonal, organizational and community (“policy level factors were not found”). |
| Cecchini et. al. 2019 *(10)* | Defined a state of avoidance-based coping |
| Coursey et. al. 2022 *(11)* | **Socio-ecological model** used in planning interview guides. Specific probes around barriers and facilitators at varying levels of the socio-ecological model (individual, intrapersonal, community, facility)  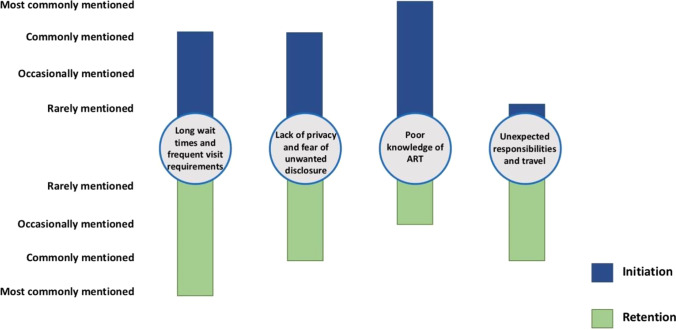 |
| Enane et. al. 2021 *(12)* | 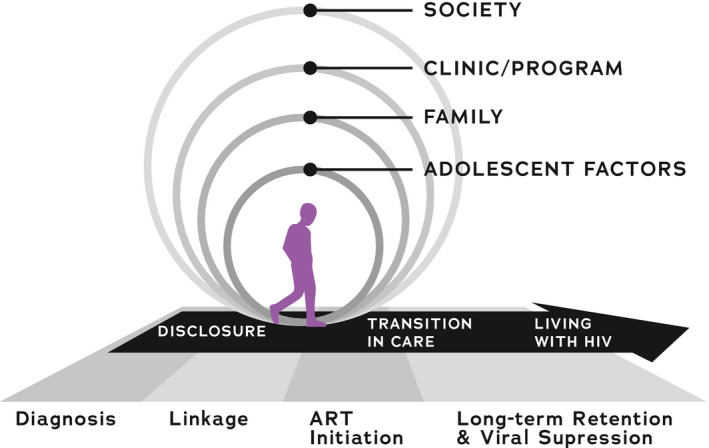 |
| Gabster et. al. 2022 *(13)* | **Explicit Social-Ecological Model -** facilitators and barriers to ART adherence and retention in care at the individual, social and structural levels.  Individual:  -Lifestyle choices  -Psychological health  Social  -Family  -Community  Structural  -Friction between Western/Traditional systems  -Food security  -Medication shortages  -Difficulties accessing ART clinic due to distance and cost |

**4. Study quality assessment**

*Approach to evidence quality assessment*

The aim of this research is to systematically review the reasons that people who have experienced disengagement from ART care report as why they disengaged.

We have included studies that report quantitative measures (typically “select multiple” type questionnaires) and studies that report on qualitative findings (typically in depth interviews and sometimes focus groups); including studies which use both methods. These methods tend to have different strengths and weaknesses. For example a quantitative study may report on how representative people surveyed are to a wider population and allow frequencies of reasons reported to be collated; by contrast qualitative studies rarely aim for representativeness and aren’t best assessed by quality of reporting frequency counts of what is mentioned. By contrast, a qualitative study may offer respondents the opportunity to explain how various reasons and factors overlap, interrelate and the relative importance of difference reasons for how they came to disengage from care; the interplay and relative import of overlapping reasons is not typically captured in a “select multiple” questionnaire.

We therefore used the following method / principles to assess study quality for this review.

- We have adapted domains from Newcastle Ottowa Scale and Critical Appraisal Skills Programme Qualitative assessment matrix.
- We apply all domains to all studies – to illustrate the issues relating to quality and bias and because some studies are mixed methods.
- We have not given an overall “score” because this could be misleading given the variety of study designs included.

These assessments should not be seen as intrinsic to quality of study but specific to our review question; survey based studies will score badly in items related to depth of understanding (typically, studies did not set out to gather deep understanding), and in depth interviews badly in representativeness (typically, these studies don’t set out to be representative).

| **Study** | **NOS / CASP: Representativeness, recruitment, selection (a)** | **NOS / CASP: Data collection / assessment of outcome (b)** | **CASP: Data analysis (c)** | **Overall (d)** |
| --- | --- | --- | --- | --- |
| Bisnauth et. al. 2021 *(1)* | People re-starting ART identified, and asked to voluntarily complete questionnaire. In depth interview participants selected to ensure representative sample.  By design, this study only includes people who have re-engaged after disengagement. Otherwise No concerns | No information on how questionnaire items developed.  No concerns re. interview guide and conduct. | Thematic analysis using conceptual framework (health system and patient factors, and changing life circumstances). Questionaires by frequency of reasons.  No concerns | **No information about how questionnaire developed / validity.**  **Otherwise no concerns.** |
| Bikokye Kafeero et. al. 2020 ABSTRACT *(14)* | Minimal information, note that of 36,702 people attempted to trace, only 5008 contacted. | Questionnaires used “customised tool”, around root cause analysis of disengagement.  Minimal information | Bar chart summarises frequency of responses. | **Minimal information on recruitment, representativeness or how tool was developed – this is a conference abstract only.** |
| Chamberlin et. al. 2021 *(2)* | Sampling strategy to recruit men / women and rural / urban.  For our definition, their definition of at least 2 weeks late to appointment is quite “permissive” definition to disengagement. Otherwise no concerns re. recruitment strategy. | In-depth interview guide taking narrative approach developed with community members.  No concerns | Analysed using domains from interview guide.  Good / no concerns | **No concerns, except that their definition of “disengagement” is quite permissive (>2 weeks late to appointment).** |
| Nsoh et. al. 2021 *(3)* | Not enough information about how participants recruited for questionnaire or IDI. | Minimal information about topic guide for IDI and no information on how questionnaire items arrived at or validated. | Themes and codes defined by interview guide and applied to transcripts of IDI. Frequency tables for survey data.  No concerns | **Minimal information about selection of participants and about how interview guide and questionnaire items arrived at.** |
| Shabalala et. al. 2018 *(4)* | Recruited based on information from MaxART study database. Only ten people interviewed.  No concerns | Flexible interviews following interview guide by experienced researchers.  No concerns | “Data-driven inductive thematic approach” to analysis.  Good / no concerns | **A relatively small number of respondent, otherwise no concerns.** |
| Atanga et. al. 2017 *(15)* | Attempted to contact women lost to follow up from PMTCT clinic. 50% of women attempted for be traced were found to ask about reasons.  No concerns. | No information, presumably some sort of questionnaire used. | Frequency of reasons in a sentence in results. | **Minimal information about how reasons obtained.** |
| Gugsa et. al. 2017 *(16)* | Women identified by routine care tracing team, and asked to enrol in study. No information about how many attempted tracing to find participants / how representative. | Semi-structured interviews using a topic guide.  No concerns. | Themes and codes inductively decided from transcripts. Frequency of reasons also reported.  No concerns. | **Unclear how representative people recruited are of all disengaged people, but otherwise no concerns**. |
| Hoffman et. al. 2017 *(17)* | Disengaged women from clinics contacted with standard tracing, then asked to enrol in study. 17% of people attempted to be traced actually found.  Does not represent all disengaged population. | No information about how reasons gathered. | Reported as frequencies. | **Minimal information to judge methods, most people not able to be traced.** |
| Kiragga et. al. 2021 *(18)* | These are people who have disengaged once, then returned to care and joined a study for ART re-starters, and then have disengaged from that study.  Slightly unusual population. | No information about how reasons gathered. | Frequency of reasons in a sentence in results. | **Minimal information about how reasons obtained.** |
| Kisigo et. al. 2020 *(5)* | Recruited based on clinic records. 42 people traced, and 12 found and agreed to participate.  Does not represent all disengaged population. | Semi-structured interviews using a topic guide.  No concerns. | Inductive thematic analysis. No concerns. | **Recruitment process well described, but a lot of attrition as people not able to be contacted. Otherwise no concerns.** |
| Kiwanuka et al. 2018 *(19)* | Disengaged women traced using standard procedures and 50% found. Then 12 women from all those traced purposely sampled for in depth interview.  Relatively small number respondents. | Semi-structured interviews using a topic guide.  No concerns. | Thematic analytic approach / development not well described. Frequency of each reason also reported. | **Relatively small number respondents. Analysis not well described.** |
| Nalubega et. al. 2021 *(6)* | All participants in a parent study. Those LTFU identified and purposive sampling to select interviewees. 17/20 people selected agreed to interview. | Semi-structured interviews using a topic guide.  No concerns. | Thematic analysis based on social-ecological model.  No concerns. | **Small study (17 people, only 9 disengagers). Otherwise, no concerns.** |
| Sariah et. al. 2019 *(7)* | Women traced via usual clinic tracing process and then invited to be in study. No information on how many women were attempted to be traced vs. how many found and agreed to participate. | Semi-structured interviews using a topic guide.  No concerns. | Inductive thematic analysis. No concerns. | **Minimal information to judge recruitment. Otherwise, no concerns.** |
| Sasse et. al. 2022 *(8)* | Recruited at point of re-entering antenatal care (in a second pregnancy, identifying people who had disengaged during/after first pregnancy).  Special population, all women who are pregnant again and seeking antenatal care. | Free form answers in a questionairre. | Eight categories developed by one researcher from responses, then remaining responses coded by other researchers. | **Some concerns about methods of obtaining reasons and slightly unusual population.** |
| Aguilera-Mijares et. al. 2022 *(9)* | Clinical staff identify people in clinic (either currently disengaged or at re-entry). Numbers contacted to recruit not well described. | Semi-structured interviews.  No concerns. | Interview transcripts coded using ecological model.  No concerns. | **Recruitment not well described, otherwise no concerns.** |
| Cecchini et. al. 2019 *(10)* | Women with adherence challenges enrolled in parent COPA2 study. | One question amid study baseline procedures about reasons for disengagement – closed question with six possible answers. Validity not described. Some concerns. | Frequency table of results. | **Some concerns about question / answer choice development.** |
| Coursey et. al. 2022 *(11)* | Men who were late to appointments traced in community by usual care staff. Numbers not described. Tracing starts when people >14 days late to appointment, a more permissive definition than ours. | Semi-structured interviews with topic guide based on ecological model. | Themes developed based on responses and existing literature, results include frequency of how often responses given. | **Minimal information about recruitment and overly permissive definition of disengagement. Otherwise no concerns.** |
| Enane et. al. 2021 *(12)* | Clinic does tracing of disengaged participants. Purposive selection from that to have balance genders. | Semi-structured interview, topic guide includes a priori topics about trauma and psychological distress as well as more general topics. | Themes developed using triangulation, consensus in research team and emergent themes – against a background of a topic guide that including probes about trauma. | **Themes around trauma developed a priori. No concerns.** |
| Kandlen et. al. 2020 ABSTRACT *(20)* | Unclear, all are people engaging with a specific programme. | Unclear. | Reports frequency in a sentence. | **Minimal information / some concerns.** |
| Gabster et. al. 2022 *(13)* | Selected by clinicians from their cohort of participants in rural / isolated area. | Semi-structured interviews. | Themes developing using socio-ecological framework, codebook in paper. No concerns. |  |

1. NOS questions in this domain are:

“Representativeness of the exposed cohort”

“Selection of the non exposed cohort”

CASP questions in this domain:

“Was the recruitment strategy appropriate to the aims of the research?”

1. NOS question in this domain is:

“Assessment of outcome” (i.e. how were reasons assessed)

CASP question in this domain:

“Was the data collected in a way that addressed the research issue?

1. CASP question: “Was the data analysis sufficiently rigorous?”
2. Specifically, as it relates to this review question – which is not necessarily the primary aim of the study.

**5. PRISMA Checklist**

| **Section and Topic** | **Item #** | **Checklist item** | **Location where item is reported** |
| --- | --- | --- | --- |
| **TITLE** | | |  |
| Title | 1 | Identify the report as a systematic review. | Title page |
| **ABSTRACT** | | |  |
| Abstract | 2 | See the PRISMA 2020 for Abstracts checklist. | Abstract |
| **INTRODUCTION** | | |  |
| Rationale | 3 | Describe the rationale for the review in the context of existing knowledge. | p. 3 |
| Objectives | 4 | Provide an explicit statement of the objective(s) or question(s) the review addresses. | p. 3 |
| **METHODS** | | |  |
| Eligibility criteria | 5 | Specify the inclusion and exclusion criteria for the review and how studies were grouped for the syntheses. | p.4 (p.6 for grouping) |
| Information sources | 6 | Specify all databases, registers, websites, organisations, reference lists and other sources searched or consulted to identify studies. Specify the date when each source was last searched or consulted. | p.4 |
| Search strategy | 7 | Present the full search strategies for all databases, registers and websites, including any filters and limits used. | Appendix |
| Selection process | 8 | Specify the methods used to decide whether a study met the inclusion criteria of the review, including how many reviewers screened each record and each report retrieved, whether they worked independently, and if applicable, details of automation tools used in the process. | p.5 |
| Data collection process | 9 | Specify the methods used to collect data from reports, including how many reviewers collected data from each report, whether they worked independently, any processes for obtaining or confirming data from study investigators, and if applicable, details of automation tools used in the process. | p.5 |
| Data items | 10a | List and define all outcomes for which data were sought. Specify whether all results that were compatible with each outcome domain in each study were sought (e.g. for all measures, time points, analyses), and if not, the methods used to decide which results to collect. | p. 5/6 |
|  | 10b | List and define all other variables for which data were sought (e.g. participant and intervention characteristics, funding sources). Describe any assumptions made about any missing or unclear information. | p. 5 |
| Study risk of bias assessment | 11 | Specify the methods used to assess risk of bias in the included studies, including details of the tool(s) used, how many reviewers assessed each study and whether they worked independently, and if applicable, details of automation tools used in the process. | p. 6 and appendix for details. |
| Effect measures | 12 | Specify for each outcome the effect measure(s) (e.g. risk ratio, mean difference) used in the synthesis or presentation of results. | NA – no effect measure. |
| Synthesis methods | 13a | Describe the processes used to decide which studies were eligible for each synthesis (e.g. tabulating the study intervention characteristics and comparing against the planned groups for each synthesis (item #5)). | NA |
|  | 13b | Describe any methods required to prepare the data for presentation or synthesis, such as handling of missing summary statistics, or data conversions. | p. 5/6 |
|  | 13c | Describe any methods used to tabulate or visually display results of individual studies and syntheses. | p. 5/6 |
|  | 13d | Describe any methods used to synthesize results and provide a rationale for the choice(s). If meta-analysis was performed, describe the model(s), method(s) to identify the presence and extent of statistical heterogeneity, and software package(s) used. | p. 6 |
|  | 13e | Describe any methods used to explore possible causes of heterogeneity among study results (e.g. subgroup analysis, meta-regression). | NA (although addressed in discussion) |
|  | 13f | Describe any sensitivity analyses conducted to assess robustness of the synthesized results. | NA |
| Reporting bias assessment | 14 | Describe any methods used to assess risk of bias due to missing results in a synthesis (arising from reporting biases). | NA |
| Certainty assessment | 15 | Describe any methods used to assess certainty (or confidence) in the body of evidence for an outcome. | p. 6 and appendix |
| **RESULTS** | | |  |
| Study selection | 16a | Describe the results of the search and selection process, from the number of records identified in the search to the number of studies included in the review, ideally using a flow diagram. | Figure 1 |
|  | 16b | Cite studies that might appear to meet the inclusion criteria, but which were excluded, and explain why they were excluded. | p. 12/13 (discussion) |
| Study characteristics | 17 | Cite each included study and present its characteristics. | Table 1 and p. 6 |
| Risk of bias in studies | 18 | Present assessments of risk of bias for each included study. | Appendix (and p. 12) |
| Results of individual studies | 19 | For all outcomes, present, for each study: (a) summary statistics for each group (where appropriate) and (b) an effect estimate and its precision (e.g. confidence/credible interval), ideally using structured tables or plots. | Table 2 (no summary statistics) |
| Results of syntheses | 20a | For each synthesis, briefly summarise the characteristics and risk of bias among contributing studies. | p. 12 |
|  | 20b | Present results of all statistical syntheses conducted. If meta-analysis was done, present for each the summary estimate and its precision (e.g. confidence/credible interval) and measures of statistical heterogeneity. If comparing groups, describe the direction of the effect. | NA (addressed in discussion) |
|  | 20c | Present results of all investigations of possible causes of heterogeneity among study results. | NA |
|  | 20d | Present results of all sensitivity analyses conducted to assess the robustness of the synthesized results. | NA |
| Reporting biases | 21 | Present assessments of risk of bias due to missing results (arising from reporting biases) for each synthesis assessed. | NA |
| Certainty of evidence | 22 | Present assessments of certainty (or confidence) in the body of evidence for each outcome assessed. | Appendix |
| **DISCUSSION** | | |  |
| Discussion | 23a | Provide a general interpretation of the results in the context of other evidence. | p. 12/13 |
|  | 23b | Discuss any limitations of the evidence included in the review. | p. 13-15 |
|  | 23c | Discuss any limitations of the review processes used. | p. 13-15 |
|  | 23d | Discuss implications of the results for practice, policy, and future research. | p. 14 |
| **OTHER INFORMATION** | | |  |
| Registration and protocol | 24a | Provide registration information for the review, including register name and registration number, or state that the review was not registered. | Appendix |
|  | 24b | Indicate where the review protocol can be accessed, or state that a protocol was not prepared. | Appendix |
|  | 24c | Describe and explain any amendments to information provided at registration or in the protocol. | NA |
| Support | 25 | Describe sources of financial or non-financial support for the review, and the role of the funders or sponsors in the review. | p. 17 |
| Competing interests | 26 | Declare any competing interests of review authors. | p. 17 |
| Availability of data, code and other materials | 27 | Report which of the following are publicly available and where they can be found: template data collection forms; data extracted from included studies; data used for all analyses; analytic code; any other materials used in the review. | p. 17 |

**6. References**

1. Bisnauth MA, Davies N, Monareng S, et al. Why do patients interrupt and return to antiretroviral therapy? Retention in HIV care from the patient’s perspective in Johannesburg, South Africa. *PloS One*, 2021, 16(9):e0256540.

2. Chamberlin S, Mphande M, Phiri K, Kalande P, Dovel K. How HIV Clients Find Their Way Back to the ART Clinic: A Qualitative Study of Disengagement and Re-engagement with HIV Care in Malawi. *AIDS and behavior*, 2022, 26(3):674–685.

3. Nsoh M, Tshimwanga KE, Ngum BA, et al. Predictors of antiretroviral therapy interruptions and factors influencing return to care at the Nkolndongo Health District, Cameroon. *African Health Sciences*, 2021, 21(Suppl):29–38.

4. Shabalala FS, Vernooij E, Pell C, et al. Understanding reasons for discontinued antiretroviral treatment among clients in test and treat: a qualitative study in Swaziland. *Journal of the International AIDS Society*, 2018, 21(Suppl Suppl 4):e25120.

5. Kisigo GA, Ngocho JS, Knettel BA, Oshosen M, Mmbaga BT, Watt MH. “At home, no one knows”: A qualitative study of retention challenges among women living with HIV in Tanzania. *PLoS ONE*, 2020, 15(8):e0238232.

6. Nalubega S, Kyenkya J, Bagaya I, et al. COVID-19 may exacerbate the clinical, structural and psychological barriers to retention in care among women living with HIV in rural and peri-urban settings in Uganda. *BMC infectious diseases*, 2021, 21(1):980.

7. Sariah A, Rugemalila J, Protas J, et al. Why did I stop? And why did I restart? Perspectives of women lost to follow-up in option B+ HIV care in Dar es Salaam, Tanzania. *BMC Public Health*, 2019, 19(1):1–11.

8. Sasse SA, Harrington BJ, DiPrete BL, et al. Factors associated with a history of treatment interruption among pregnant women living with HIV in Malawi: A cross-sectional study. *PloS One*, 2022, 17(4):e0267085.

9. Aguilera-Mijares S, Martínez-Dávalos A, Vermandere H, Bautista-Arredondo S. HIV Care Disengagement and Antiretroviral Treatment Discontinuation in Mexico: A Qualitative Study Based on the Ecological Model Among Men Who Have Sex With Men. *Journal of the Association of Nurses in AIDS Care*, 2022, 33(4):468.

10. Cecchini D, Alcaide ML, Rodriguez V de J, et al. Women of Reproductive Age Living with HIV in Argentina: Unique Challenges for Reengagement in Care. *Journal of the International Association of Providers of AIDS Care*, 2019, 18:2325958219883250.

11. Coursey K, Phiri K, Choko AT, et al. Understanding the Unique Barriers and Facilitators that Affect Men’s Initiation and Retention in HIV Care: A Qualitative Study to Inform Interventions for Men Across the Treatment Cascade in Malawi. *AIDS and behavior*, 2023, 27(6):1766–1775.

12. Enane LA, Apondi E, Omollo M, et al. ‘I just keep quiet about it and act as if everything is alright’ - The cascade from trauma to disengagement among adolescents living with HIV in western Kenya. *Journal of the International AIDS Society*, 2021, 24(4):e25695.

13. Gabster A. Socha E. Pascale J. M. Talavero G. C. Castrellon A. Quiel Y. Gantes C. Mayaud P. Barriers and facilitators to antiretroviral adherence and retention in HIV care among people living with HIV in the Comarca Ngabe- Bugle, Panama. *PLoS ONE.*, 2022, 17(6):NA.

14. Bikokye Kafeero. Root cause analysis as quality improvement tool for identification of barriers to improve retention in HIV care: The case of Uganda. *Journal of the International AIDS Society.*, 2020:23rd International AIDS Conference. Virtual. 23(SUPPL 4) (no pagination).

15. Atanga PN, Ndetan HT, Achidi EA, Meriki HD, Hoelscher M, Kroidl A. Retention in care and reasons for discontinuation of lifelong antiretroviral therapy in a cohort of Cameroonian pregnant and breastfeeding HIV-positive women initiating ‘Option B+’ in the South West Region. *Tropical medicine & international health: TM & IH*, 2017, 22(2):161–170.

16. Gugsa S, Potter K, Tweya H, et al. Exploring factors associated with ART adherence and retention in care under Option B+ strategy in Malawi: A qualitative study. *PLOS ONE*, 2017, 12(6):e0179838.

17. Hoffman RM, Phiri K, Parent J, et al. Factors associated with retention in Option B+ in Malawi: a case control study. *Journal of the International AIDS Society*, 2017, 20(1):21464.

18. Kiragga AN, Twinomuhwezi E, Banturaki G, et al. Outcomes of retained and disengaged pregnant women living with HIV in Uganda. *PLOS ONE*, 2021, 16(5):e0251413.

19. Kiwanuka G, Kiwanuka N, Muneza F, et al. Retention of HIV infected pregnant and breastfeeding women on option B+ in Gomba District, Uganda: a retrospective cohort study. *BMC Infectious Diseases*, 2018, 18(1):533.

20. Kandlen, K. Comprehensive retention to care model: Results of the pilot in Krasnoyarsk, Russia 2016 to 2019. *Journal of the International AIDS Society.*, 2020:HIV Glasgow. Virtual. 23(SUPPL 7) (no pagination).
